# Selections

**Published:** 1893-12

**Authors:** 


### Selections.

### A STATEMENT OF SOME FACTS IN REGARD TO THE ORIGIN OF THE PRACTICAL APPLICATION OF ANÆSTHESIA IN SURGERY.

BOSTON, October 2, 1893.

THAT sulphuric ether was repeatedly used as an anæsthetic by Dr. Long, of Athens, Georgia, in his surgical practice as early as 1840 seems indisputable from the reliable evidence collected and published by the late Dr. Marion Sims, of New York, but a few years ago.

The fact that Dr. Long did not publish it to the world does not affect the priority of its use.

Next comes, about four or five years later, the experimental discovery and practical application by Dr. Wells, of Hartford, Connecticut, of the anæsthetic properties of nitrous oxide gas.

Then come facts known to me personally, as I was "behind the scenes." About a year or more later, Dr. Morton, remembering vaguely that Dr. Wells had faith in anæsthesia by the inhalation of something, applied to Dr. Jackson with the inquiry if there was anything that could be so used. Dr. Jackson informed him that in his personal experience sulphuric ether had relieved his suffering from the accidental breathing of chlorine gas, adding a caution in regard to its use. Dr. Morton, finding Dr. Jackson's information correct in regard to the "letheal" effects of sulphuric ether, by persuasion induced Dr. Jackson to unite with him in procuring a patent to control its use. Dr. Jackson's medical friends reminding him of the breach of ethics in getting such a patent, he withdrew from the patent. It was *then* that Dr. Morton claimed the sole discovery of ether anæsthesia, and, wishing to have some other respectable backing, he went to Dr. John C. Warren, then the head of the surgical staff of the Massachusetts General Hospital, and said, in his persuasive way, that he had discovered a "compound," which he called "letheon," that would prevent pain in surgical operations, and would be pleased to show its effects on some hospital patient.

Dr. Warren consented to the trial, which proved successful; but he, with the rest of the staff, declined to adopt or endorse it unless they knew what it actually was. Of course, Dr. Morton had to tell them that it was sulphuric ether. They then began its use; and it was *their endorsement* which gave it the successful start in the surgical world, without which it is very probable it might have fallen into the list of empirical fancies.

The summing up of these facts seems like this: First, Dr. Long's successful private practice, followed after several years by Dr. Wells's success, which his diffident nature hindered him from pushing to notoriety. Then Dr. Jackson's instruction to Dr. Morton, which he followed out and brought to the notice of Dr. Warren and the surgical staff of the Massachusetts General Hospital; which staff, having eliminated its elements of quackery, tried effectually, and with their substantial endorsement gave it to the world. To that *endorsement*, it seems, is due the permanent consideration that anæsthesia has since received.

JACOB L. WILLIAMS, M.D.

—*Boston Medical and Surgical Journal.*

---

### THE HYDROGEN DIOXIDE CONTROVERSY.

AN acrid controversy in regard to the availability of hydrogen dioxide (commonly called peroxide of hydrogen), has arisen between a prominent New York physician and a manufacturer of this product, which seems to call for brief comment, since the writer has been using it continuously for the past seven years. His first published paper appeared September 1, 1888 (Philadelphia *Medical Times*), and his conclusions, therefore, may be accepted as representing the results of clinical observation during that period.

Hydrogen dioxide is relatively, but not absolutely, harmless, pointed out as long ago as 1862, by Dr. Benjamin Ward Richardson. Like mercury, it will cause the teeth to become loose, and that the writer has witnessed in the case of a patient who deemed himself capable of self-medication; but a freshly-prepared product, free from an excess of acid and other impurities, properly used, is absolutely harmless. In suitable strength—one part to six to ten of the fifteen-volume solution—it is a useful and efficient remedy in diphtheria,—that is, a solution conforming to the above requirements. Not being a stable article, a solution which is reliable to-day may be worthless, irritating, or even poisonous to-morrow. Any solution when combined with a comparatively pure glycerin and allowed to stand, will produce an irritant action similar to formic acid, but this is not due to the faulty character of the dioxide; it is owing to the chemical changes resulting from oxidation.

In the treatment of nasal catarrh occurring in debilitated subjects, where the tissues are relaxed and "juicy," a weak solution causes burning and smarting, although it removes every vestige of accumulated mucus. To overcome this, it is advisable to employ a petroleum spray, plain or suitably medicated, or, in the absence of an atomizer, a colorless petroleum ointment may be substituted.

In diphtheria the same course is pursued, but we must bear in mind that while diphtheria is at first a local affection, poisonous products are rapidly absorbed from the seat of disease, so that in the course of a few hours it presents all the symptoms of constitutional infection. Internal medication is imperatively demanded to counteract the toxic action of the pathogenic micro-organism associated with the disease, over which local treatment has no influence whatever.

Knowing the exceedingly vascular character of the nasal mucous structures and the large amount of moisture eliminated by them, we do not have far to go for an explanation of the bad effects following the use of hydrogen dioxide. The fault rests entirely with internal medication, because thorough cleansing of the diseased area only opens the sluice-way for increased elimination of poisons, and we have a clinical paradox. The efficacy of the remedy is its only disadvantage.—*Editorial in American Therapist.*

---

### RESEARCHES IN BACTERIOLOGY.

To-day bacteriology is about to revolutionize medicine by elaborating a specific treatment of the infectious diseases.

The demonstration that pathogenic bacteria are virulent on account of their chemical products, and the study of the toxines and toxalbumens of infectious diseases, led to the discovery of antitoxines in the blood of animals immune against certain diseases, and these antitoxines were found to have an unexpected antidotal power. The researches of Büchner, Martin, Hankin, Nuttall, and others paved the way for the demonstration by Ogata and Jashuhara that the injection of a drop of blood from an immune frog will protect a mouse against an ordinary fatal anthrax inoculation, and then Behring and Kitasato showed that the blood of animals immune against tetanus and diphtheria, if injected into susceptible animals, prevents fatal infection with virulent cultures of the bacilli of these diseases; suitable experiments readily showed that the toxalbumen of the tetanus bacillus is neutralized when mixed with the blood of immune animals, and the next step was the isolation of the tetanus-antitoxine by Tizzoni and Cantani and the successful treatment of actual cases of traumatic tetanus by means of tetanus-antitoxine injections. Already six cases successfully treated by this novel and specific but absolutely scientific method have been recorded in medical literature, the first one being reported by Rudolph Schwartz.

G. and F. Klemperer then showed that the blood-serum of animals artificially immune against croupous pneumonia renders susceptible animals immune, and that it has a direct curative effect if injected after the development of the disease. Preliminary communications are at hand announcing the discovery of antitoxines antidotal to tuberculosis and to rabies, and numerous ingenious experiments are constantly being made demonstrating that acquired immunity is due to the development of antitoxines. Recently Sternberg has demonstrated by proper experiments that the blood-serum of calves immune by previous attacks of vaccinia to vaccine virus contains something which neutralizes humanized or bovine lymph.—*Editorial in Journal American Medical Association.*

---

### TO DEODORIZE IODOFORM, CREOSOTE, AND GUAIACOL.

The odor of iodoform, creosote, or guaiacol upon the hands can be overcome by washing with linseed meal. Articles having an odor of iodoform may be washed in tar-water to which oil of wintergreen has been added. The taste of pills of creosote can be disguised by means of a little powdered coffee. The odor of iodoform or guaiacol in rooms can be dissipated by burning coffee.—*Deutsche Medizinal Zeitung* (*Medical News*).

### PHENOCOLL HYDROCHLORIDE AS A LOCAL APPLICATION.

A PAPER by Dr. Carl Beck, in the *New York Medical Journal*, April 22, presents an argument in favor of the antiseptic external use of the drug named in the caption. He finds in this drug a good substitute for iodoform, probably as powerful as the latter, and rather more so than aristol, dermatol, iodol, pyoktanin, and some others that have been coming to us from Germany during the past year. Apart from the question of strength, the author prefers phenocoll to iodoform for the following reasons: the former is devoid of odor; it is readily soluble in hot water; it does not irritate the sound skin; it is not contra-indicated in cases of kidney-disease; it can be safely applied over extensive surfaces, as of burns or ulcers; it is potent in comparatively low percentages of strength. When Dr. Beck began the external use of this substance, he dusted the undiluted powder over the wounded surfaces and then applied a layer of sterilized moss or gauze. Although this treatment was followed by no irritation of the integument and by no symptoms of toxic impression, he found that he obtained equally good results, in many cases, from a ten-per-cent. gauze; so that latterly he has limited himself to the use of the phenocollated gauze. This can be used on recent wounds and on granulating surfaces; the layer of gauze should be thin and as a rule be protected by a piece of sterilized moss. The dressing may be renewed once in three days, for there is not an excessive discharge from surfaces thus managed. The process of healing does not vary much from that observed under iodoform. The urine was frequently examined in the case of each one of this series of cases, numbering over one hundred, with negative results as to the discovery of any renal disturbance referable to the drug. In fact, albuminous urine has not very frequently been caused by its internal administration as an antipyretic or antirheumatic, which have heretofore been the best known uses of phenocoll hydrochloride.

The report of Dr. Beck covers the experience of three months at St. Mark's Hospital and the German Poliklinik of New York, and is the first instalment of researches that are still being carried out with regard to the antiseptic powers of the drug named, as well as others of the synthetic series.—*Journ. Amer. Med. Assoc.*

